# Donglin Jiang answers questions about 15 years of research on covalent organic frameworks

**DOI:** 10.1038/s41467-020-19302-x

**Published:** 2020-11-16

## Abstract

Prof. Donglin Jiang is a full professor at the National University of Singapore and is recognized as a pioneer in the field of 2D polymers and covalent organic frameworks (COFs). With a background in chemistry and studies on dendrimers for ten years, the beauty of dendrimers inspired him to consider the possibility of constructing other types of polymers with well-defined shapes and structures. After taking up an associate professorship in 2005 to set up an independent laboratory at the Institute for Molecular Science at the National Institute for Natural Science, he started to dedicate his work on the design, synthesis and functional exploration of 2D polymers, COFs and conjugated microporous polymers.

Tell us a little bit about your research background and how you became interested in the chemistry of porous materials and COFs.

As a chemist I have worked on the synthesis and functional exploration of dendrimers since 1995 at the University of Tokyo and from that time I became very interested in polymers with well-defined structures. The beautiful shape of dendrimers inspired me to consider the possibility of constructing other types of polymers with well-defined shapes and structures. In 2005 I moved to the Institute for Molecular Science at the National Institute for Natural Sciences and I developed supramolecular polymers that have self-assembled 2D sheet structures and stacked to form layer structures. From that study I got inspired on whether I can develop 2D polymers that are covalently linked. We succeeded in finding the phenazine linkage to covalently connect π building units into stable 2D polymers and layer frameworks via one-pot polymerization. This was the starting point where we involved in developing COFs by our own strategy and ideas.

Looking back on 15 years research on COFs, what were the highlights in COF research and which expectations remained unfulfilled?

Personally I think that COFs are a new and amazing class of materials that bridge the gap between organic chemistry and materials science. The most attractive feature is that COFs enable the predesign of both primary- and high-order structures. For traditional polymers, we can design primary-order chain structures by controlled or living polymerization, but we hardly design their high-order structures as polymer chains aggregate to form disordered materials. This has been a long-term challenge in polymer science. COFs make it possible as they merge both covalent bonds and noncovalent interactions into one polymerization system. The covalent bonds develop ordered polymeric skeletons (primary-order structure) while the noncovalent interactions control folding or stacking to construct frameworks (high-order structure). I think one distinct feature is that COFs allow us to construct both primary- and high-order structures in a predetermined way.

I think the field has already exerted a lot of impacts on chemistry. We have now a high freedom to design polymers and organic materials with long-range ordered structures. From the very beginning we could not envision what the possibilities of COF structures are and after 15 years there are many clear visions and new findings which we have explored. For example, we designed well-defined skeletons with periodically ordered π columns, which enable us to establish a new type of organic semiconductors. These systems form topologically defined π architectures with the ability to form unidirectionally orientated π arrays. These semiconductors are unique compared to assemblies of conventional polymers and even single crystals of organic π compounds. I love the idea to develop COFs as functional porous materials. This is another distinct feature of COFs where we can design the pore shape and size and we even constructed the pore interface in a tailor-made way. These predesignable pore parameters control the interaction with molecules and ions and thus determine the property of pores, such as molecular storage or mass transport. We employed a complementary way to develop molecular systems for energy conversion and storage by designing the skeleton and pores to connect the involved chemical and physical processes into a seamless chain of events.

In your opinion, which challenges need to be tackled in order to move the field further?

I think there are still a lot of fundamental issues which need to be addressed in future. From a synthesis point of view, I think the key is to decrease the hurdles to access high quality framework materials or to develop a general way to produce single crystals. From physics perspective, I think the important issue is to understand the chemical or physical events at different time and spatial scales. Especially, disclosing interplays of COFs with photons, excitons, phonons, electrons, holes, ions, and molecules to identify their nature is a key point, which will lead to the findings of new phenomena and mechanisms. From materials science perspective, I believe the exploration of properties and functions that are specific to the structures of COFs is a major challenge in the future. I think COFs need to develop unique property and function which are inaccessible with other polymers and framework materials. In my view, this point is determining the impact of the COF materials and will definitely lead to large scale applications.

What needs to be done in order to make COFs more widely adopted and to get a foothold in industry and industrial applications?

I think that COFs have already established as a new class of polymers or molecular frameworks. I think it is highly possible that COFs see an uptake from industry because this system is based on a clean polymerization system to synthesise and is basically scalable. The question is which functions are truly unique or specific to COFs and can be developed for large scale applications. As example: COFs can be used for photoconversion or reducing carbon emission.

Where do you see the field going next?

I think while the field has made a great progress to achieve the diversity of structures and functions, and some unique structures and functions could be developed next. I think one of big things is to explore quantum phenomena by designing specific π electronic structures. Another one is related to the pores to realize unusual storage and transporting properties and functions. By complementary use of the skeleton and pore, designing unique molecular systems that connect various chemical and physical processes can be expected. I envision that with these further progresses, we will have the chance to disclose their hidden nature and to show their important role in addressing environmental and energy problems.

*The interview was conducted by Senior Editor Dr. Johannes Kreutzer*.

